# Clinical Risk Factors for Death in Patients With Empyema and Active Malignancy

**DOI:** 10.7759/cureus.37545

**Published:** 2023-04-13

**Authors:** Moiz Salahuddin, David Ost, Hyunsoo Hwang, Carlos Jimenez, Sahara Saltijeral, George Eapen, Roberto Casal, Bruce Sabath, Julie Lin, Eben Cerrillos, Tamara Nevárez Tinoco, Horiana Grosu

**Affiliations:** 1 Pulmonology and Critical Care, Aga Khan University Hospital, Karachi, PAK; 2 Pulmonary Medicine, Monroe Dunaway (MD) Anderson Cancer Center, Houston, USA; 3 Biostatistics and Epidemiology, Monroe Dunaway (MD) Anderson Cancer Center, Houston, USA; 4 Obstetrics and Gynecology, Monroe Dunaway (MD) Anderson Cancer Center, Houston, USA; 5 Internal Medicine, Instituto Tecnologico y de Estudios Superiores de Monterrey, Monterrey, MEX

**Keywords:** malignant pleural effusion, thoracic empyema, pleural disease, pleural empyema, pleural effusion

## Abstract

Background

Pleural infection is a common clinical problem resulting in prolonged hospitalization and increased mortality. In patients with active malignancy, management decisions are based on the need for further immunosuppressive therapies, the ability to tolerate surgery, and consideration of the limited life expectancy. Identifying patients at risk for death or poor outcomes is very important as it will guide care.

Study design and methods

This is a retrospective cohort study of all patients with active malignancy and empyema. The primary outcome was time to death from empyema at three months. The secondary outcome was surgery at 30 days. Standard Cox regression model and cause-specific hazard regression model were used to analyze the data.

Results

A total of 202 patients with active malignancy and empyema were included. The overall mortality rate at three months was 32.7%. On multivariable analysis, female gender and higher urea were associated with an increased risk of death from empyema at three months. The area under the curve (AUC) of the model was 0.70. The risk factors for surgery at 30 days included the presence of frank pus and postsurgical empyema. The AUC of the model was 0.76.

Interpretation

Patients with active malignancy and empyema have a high probability of death. In our model, the risk factors for death from empyema included female gender and higher urea.

## Introduction

Pleural infection is a common clinical problem with an increasing incidence of six per 100,000 people [1]. The 30-day mortality for empyema is approximately 7%-11% [2]. Pathogens causing empyema in the developed world are commonly *Streptococcus* and *Staphylococcus*, whereas tuberculosis is seen more commonly in the developing world [1]. Despite medical advances, mortality is still high, and 20% of patients require surgical intervention [3]. Most patients with empyema have long hospital stays with a median of 12-21 days [3,4]. Treatment consists of antibiotics and drainage of the empyema, usually via chest tube and sometimes with surgery. More recently, intrapleural tissue plasminogen activator (tPA) combined with deoxyribonuclease (DNAse) has been used to facilitate more effective chest tube drainage of empyemas [5].

Identifying patients at risk for death or poor outcome is very important as it may direct care toward a more aggressive strategy early on such as surgery. Some models have found polymicrobial empyema to be a risk factor for in-hospital mortality [6]. More recently, the renal, age, purulence, infection source, and dietary factors (RAPID) score has been shown to allow risk stratification in patients with pleural infection at presentation and has been validated [2]. Five key prognostic features of the RAPID score at baseline were found to be predictors for overall mortality in patients with empyema [2]. The score stratifies patients into low (0-2), medium (3-4), and high (5-7) risk groups, with higher scores being associated with increased three-month overall mortality and increased hospital stay [2].

We believe that compared to empyema in patients without cancer, patients with both empyema and active cancer are different. In cancer patients, the decision to intervene surgically is often influenced by the need for further cancer chemotherapy, frequently a lower ability to tolerate surgery, and consideration of the competing risk of death given the limited life expectancy of patients with active cancer. The high background rate of death due to active cancer often forces changes in clinical management for other comorbidities. To date, however, there are no predictors for mortality in patients with active malignancy who develop empyema, and the RAPID score is not appropriate for patients with empyema and active cancer as most of these patients may not survive beyond three months [2]. Our primary objective was to quantify the cumulative incidence of death due to empyema and identify risk factors associated with death due to empyema in patients with active malignancy. Our secondary objectives were to quantify how often surgical intervention was required for empyema in patients who also had active cancer and identify factors associated with surgical intervention.

## Materials and methods

This is a retrospective review of all patients with active malignancy and empyema who were evaluated from January 1, 2005, to December 2015. Approval was obtained from Institutional Review Board Committee 4, PA2020-0619.

We used the International Classification of Diseases (ICD) 9 (ICD-9) and ICD-10 diagnostic codes for empyema. Empyema diagnosis was defined based on positive pleural fluid culture for bacteria or fungus, or aspiration of frank pus from the pleural cavity. For indwelling pleural catheter (IPC)-related empyema, patients needed to have positive cultures from a separate thoracentesis as per institutional guidelines or aspiration of frank pus via the IPC.

We included all adult patients more than 18 years of age with active cancer and a diagnosis of empyema. Active cancer was defined as those patients who are undergoing active cancer treatment such as chemotherapy, radiation, and surgery. We excluded patients who did not have evidence of empyema and those with no active cancer and those with a history of cancer who are considered in remission or “cured.” Two separate physician reviewers assessed the charts to assess for inclusion and exclusion criteria and ascertain the cause of death.

The category of healthcare-associated pneumonia (HCAP) was included in the 2005 American Thoracic Society/Infectious Diseases Society of America (ATS/IDSA) guidelines and referred to pneumonia acquired in healthcare facilities such as nursing homes, hemodialysis centers, and outpatient clinics or during hospitalization within the past three months [7]. Since our data was collected before the 2016 ATS/IDSA guidelines, we used the HCAP definition for our cohort.

Our primary outcome was death due to empyema at three months, measured as days from the diagnosis of the empyema to the day of death from empyema. Two separate physicians judged whether the patients’ cause of death was due to empyema or malignancy. Empyema death was assigned if patients developed an uncontrolled infection and complications related to the empyema that led to their death, while their malignancy was not end-stage. If patients recovered from empyema and infectious markers improved but their malignancy was progressive and end-stage, then the cause of death was assigned to be a malignancy. Our secondary outcome was to evaluate clinical worsening requiring surgery at 30 days in patients with active malignancy that developed empyema.

Data analysis

Patient characteristics were summarized using median (range) and frequency (percentage) for continuous and categorical variables, respectively. We analyzed a dataset depending on when the observations were censored. The three-month dataset censored all the observations at three months who are alive at three months.

Cox proportional hazards (PH) regression models were applied to assess the association between patient characteristics and overall survival outcomes. For time to death from empyema, which is the primary outcome, the cause-specific (empyema-specific) hazard regression model was fit by treating death from empyema as events and death from other causes as censored observation. We used this methodology to consider deaths from underlying malignancy.

A logistic regression model was also fit to assess the association between patient characteristics and the need for surgery.

Factors whose significance at 0.20 or below in the univariate models were included in the multivariable model building. Then, backward elimination was applied until all the remaining variables had p values < 0.05. The cumulative failure rates over time were estimated and represented using cumulative incidence analysis. Performances of models were compared using the area under the curve (AUC) and Brier. All statistical analyses were performed using R 4.1.1, and statistical significance was achieved at 0.05.

## Results

We identified 960 patients with a diagnosis code of empyema. However, after reviewing each chart, only 202 patients met our inclusion criteria. The median patient age was 62 years, and 130 (64.6%) patients were males. Primary lung cancer comprised 55 (27.2%) patients, solid organ non-lung cancers 105 (51.9%), and liquid malignancy 42 (20.8%). Due to the heterogeneity of our cohort, we categorized the patients into pneumonia-related empyema, IPC-related empyema, and postsurgical empyema. The source of infection was hospital-acquired pneumonia in 115 (56.9%) patients, IPC related in 51 (25.3%), and postsurgical in 36 (17.8%). Table 1 shows the details of patient characteristics. The overall mortality rate at three months was 32.7% and at 12 months was 55.9%.

**Table 1:** Patient and clinical characteristics of patients with active malignancy and empyema LDH: lactate dehydrogenase, HCAP: healthcare-associated pneumonia, IPC: indwelling pleural catheter

Patient characteristics	Median (range)/frequency (percentage)
Age (years)	62 (22, 91)
Age (years)	
21-49	35 (17.33%)
50-70	128 (63.37%)
71-91	39 (19.31%)
Gender	
Female	72 (35.64%)
Male	130 (64.36%)
Race	
Asian	6 (2.97%)
Black	21 (10.4%)
Hispanic	18 (8.91%)
Other	5 (2.48%)
White	152 (75.25%)
Cancer type	
Liquid malignancy	42 (20.79%)
Lung cancer	55 (27.23%)
Solid non-lung	105 (51.98%)
Frank pus in fluid	
No	137 (67.82%)
Yes	65 (32.18%)
Fluid LDH (U/L)	2,819 (188, 54,950)
Fluid glucose (mg/dL)	35 (15, 69)
Fluid culture	
Gram negative	27 (13.37%)
Gram positive	73 (36.14%)
No growth	83 (41.09%)
Other	19 (9.41%)
Side of empyema	
Left	87 (43.07%)
Right	115 (56.93%)
Source of infection	
HCAP	115 (56.93%)
IPC-related	51 (25.25%)
Postsurgical	36 (17.82%)
Surgery 30 days prior to diagnosis of empyema	
No	162 (80.2%)
Yes	40 (19.8%)
Chemotherapy 30 days prior to diagnosis of empyema	
No	62 (30.69%)
Yes	140 (69.31%)
Transplant status	
No	192 (95.05%)
Yes	10 (4.95%)
Chronic immunosuppression	
No	160 (79.21%)
Yes	42 (20.79%)
Radiation 30 days prior to diagnosis of empyema	
No	179 (88.61%)
Yes	23 (11.39%)

Cause-specific (empyema-specific death) model at three months

On univariate analysis, compared with lung cancer, hematologic malignancy was associated with an increased risk of death from empyema. Similarly, compared with lower urea levels, having higher urea was associated with an increased risk of death from empyema, and having a stem cell transplant status was associated with a higher risk of death from empyema. However, on multivariable analysis, female gender and higher urea were associated with an increased risk of death from empyema (Table 2). The AUC and Brier scores of the model were 0.70 (0.65; 0.76) and 8.3 (5.2; 11.4), respectively. 

**Table 2:** Empyema-specific death in patients with active malignancy at three months HR: hazard ratio, CI: confidence interval, LDH: lactate dehydrogenase, HCAP: healthcare-associated pneumonia, IPC: indwelling pleural catheter

	Univariate analysis		Multivariate analysis	
	HR (95% CI for HR)	p value	HR (95% CI for HR)	p value
Gender				
Male				
Female	1.84 (0.83-4.09)	0.14	2.55 (1.12-5.79)	0.03
Race				
White				
Others	1.54 (0.66-3.6)	0.32		
Age (years)				
21-49				
50-70	1.34 (0.46-3.93)	0.59		
71-91	0 (0-Inf)	1.00		
Cancer type				
Lung cancer				
Liquid	3.87 (1.03-14.6)	0.05		
Solid, non-lung	2.35 (0.67-8.24)	0.18		
Fluid pus				
No				
Yes	0.39 (0.13-1.14)	0.09		
Fluid LDH (U/L)	1 (1-1)	0.76		
Fluid glucose (mg/dL)	1 (0.99-1.01)	0.92		
Fluid culture				
No growth				
Gram negative	2.01 (0.66-6.15)	0.22		
Gram positive	1.41 (0.56-3.58)	0.46		
Other	0.54 (0.07-4.31)	0.56		
Urea				
<5				
5-8	2.81 (0.92-8.59)	0.07	3.2 (1.04-9.82)	0.04
>8	4.83 (1.68-13.92)	0.004	6.19 (2.1-18.26)	0.001
Albumin (g/dL)				
<27				
≥27	0.56 (0.25-1.25)	0.16		
Side of empyema				
Right				
Left	0.46 (0.2-1.07)	0.07		
Source of infection				
HCAP				
IPC	0.47 (0.16-1.37)	0.17		
Postsurgical	0.15 (0.02-1.14)	0.07		
Surgery 30 days before				
No				
Yes	0.35 (0.08-1.48)	0.15		
Chemotherapy 30 days before				
No				
Yes	1.79 (0.67-4.78)	0.25		
Chronic immunosuppression				
No				
Yes	2.06 (0.88-4.82)	0.10		
Radiation 30 days before				
No				
Yes	1.65 (0.56-4.81)	0.36		
Transplant				
No				
Yes	3.37 (1-11.36)	0.05		

Factors associated with surgery at 30 days

On univariate analysis, the odds of surgery were increased by the presence of pus (p=0.02), pleural fluid culture showing other (p=0.04), postsurgical empyema compared to hospital-acquired pneumonia (HAP) (p<0.001), and solid non-lung cancer compared to lung cancer (p=0.03). “Other” organisms were found in 19 (9.41%) patients, which comprised polybacterial in 15 patients and fungal in the remaining four patients. On multivariable analysis, the presence of pus and postsurgical empyema were associated with higher odds of surgery. The AUC of the model was 0.76.

## Discussion

This is the first study to evaluate factors associated with mortality from empyema in patients with active malignancy. Our cohort had a three-month all-cause mortality of 32.7% and a 12-month all-cause mortality of 55.9%. Prior studies of empyema reported in-hospital 30-day mortality of approximately 10%-15% and a one-year mortality rate of 20%-45% [8-14]. However, this higher mortality rate in our cohort is attributed to malignancy as the cause of death and not due to the empyema itself. In our cohort, 84 (41.5%) patients died due to their underlying malignancy. The cause of death at three months from empyema in our patient population was 11.8%, which is similar to the rates reported in the literature. Despite the heterogeneity of our cohort, there was no difference found in mortality in those patients with pneumonia-associated empyema compared to IPC-related or postsurgical empyema. In our cohort, most of the patients had hospital-acquired infections, and this is fundamentally different from the general population. Hospital-acquired pleural infection is known to carry a worse outcome and prognosis.

The predictive model showed on multivariable analysis that female gender and higher urea were associated with an increased risk of death from empyema. At three months, this model performed well with an AUC of 0.70. In addition, for the outcome of surgical intervention at 30 days, our model performed well with an AUC of 0.76. The model showed that the presence of frank pus and postsurgical empyema were associated with higher odds of surgical intervention at 30 days. This is to be expected as patients with postsurgical empyema are more likely to be taken for a reoperation as maybe these patients did not have malignant effusion such as those with an IPC in place. Also, although IPC-related empyema accounted for 25% of all empyema in this study, these patients underwent surgical intervention less commonly than postsurgical empyema.

In our cohort, 50 (24.7%) patients underwent surgery for empyema, 24/115 (20.8%) for pneumonia-related empyema, 5/51 (9.8%) for IPC-related empyema, and 21/36 (58.3%) for postsurgical empyema. There were 28 patients who underwent video-assisted thoracoscopic surgery and 22 who underwent thoracotomy. The surgical rates for empyema were lower in a New Zealand study, where 13% of patients were referred for surgery, and in the First Multicenter Intrapleural Sepsis Trial (MIST-1) trial, where the overall surgical referral rate was 14.8% [14,15]. This indicates that despite active malignancies, our cohort had relatively low thresholds to proceed with surgical management in those patients with pneumonia-related and postsurgical empyema. Nayak et al. showed that surgical intervention for thoracic empyema is associated with lower 30-day and one-year mortality compared to nonsurgical management [16]. However, we should keep in mind that the MIST-2 trial was done in 2011 and that studies that include patients before that may not have used tPA/DNAse as standard nonsurgical management of empyema, with reports of only 3%-20% of patients receiving fibrinolytics [8,9,15].

This is the first study to use a cause-specific hazard regression for patients with cancer and empyema. This is because, in our patient population, there were many patients dying from their underlying disease rather than empyema. When competing risks are present, such as in this case, mortality in patients with cancer is high and will be competing with the mortality from empyema. The Kaplan-Meier survival function will consistently overestimate the crude incidence of the outcome of interest [17].

In our cohort, the rate of pleural fluid culture positivity was 58.91%, which is quite high. Other literature suggests that in the general population and especially in pediatrics where most studies have been done, the yield is around 17%-30% [18]. A possibility that can explain this is the type of infections patients in the cohort had, including postsurgical and IPC-related infections.

We do recognize that our study has limitations, such as, firstly, the retrospective nature of data collection. Secondly, our study is a single-center cohort study, which consists only of cancer patients; therefore, it should not be applicable to the general population. Thirdly, we do not have a validation dataset for our model to predict its external validity.

## Conclusions

In conclusion, patients with active malignancy and empyema have a high probability of death at three months. The risk factors for death included female gender and higher urea. Future studies will be needed and should be designed with special attention to the type of treatment, antibiotic use, and the criteria used to justify surgery.
